# HrcQ Provides a Docking Site for Early and Late Type III Secretion Substrates from *Xanthomonas*

**DOI:** 10.1371/journal.pone.0051063

**Published:** 2012-11-30

**Authors:** Christian Lorenz, Jens Hausner, Daniela Büttner

**Affiliations:** Institute of Biology, Genetics Department, Martin Luther University Halle-Wittenberg, Halle (Saale), Germany; Centre National de la Recherche Scientifique, Aix-Marseille Université, France

## Abstract

Pathogenicity of many Gram-negative bacteria depends on a type III secretion (T3S) system which translocates bacterial effector proteins into eukaryotic cells. The membrane-spanning secretion apparatus is associated with a cytoplasmic ATPase complex and a predicted cytoplasmic (C) ring structure which is proposed to provide a substrate docking platform for secreted proteins. In this study, we show that the putative C ring component HrcQ from the plant pathogenic bacterium *Xanthomonas campestris* pv. *vesicatoria* is essential for bacterial pathogenicity and T3S. Fractionation studies revealed that HrcQ localizes to the cytoplasm and associates with the bacterial membranes under T3S-permissive conditions. HrcQ binds to the cytoplasmic T3S-ATPase HrcN, its predicted regulator HrcL and the cytoplasmic domains of the inner membrane proteins HrcV and HrcU. Furthermore, we observed an interaction between HrcQ and secreted proteins including early and late T3S substrates. HrcQ might therefore act as a general substrate acceptor site of the T3S system and is presumably part of a larger protein complex. Interestingly, the N-terminal export signal of the T3S substrate AvrBs3 is dispensable for the interaction with HrcQ, suggesting that binding of AvrBs3 to HrcQ occurs after its initial targeting to the T3S system.

## Introduction

Many Gram-negative pathogenic bacteria employ a type III secretion (T3S) system to translocate effector proteins into eukaryotic cells. T3S systems are conserved among plant and animal pathogenic bacteria and are evolutionarily related to the bacterial flagellum, which is the key bacterial motility organelle and hereafter is referred to as flagellar T3S system [1], [2], [3]. Electron microscopy studies of isolated flagellar and translocation-associated T3S systems from *Salmonella* spp. and *Shigella flexneri*, respectively, revealed that the membrane-spanning portions of both T3S systems share a similar architecture consisting of ring structures in the inner membrane (IM) and outer membrane (OM) [4]-[9]. These architectural similarities are reflected by homologies in the amino acid sequences of at least eight components of the secretion machinery that presumably constitute the core structural elements of the T3S system. In translocation-associated T3S systems the nomenclature of these proteins refers to the Ysc proteins from the animal pathogenic bacterium *Yersinia* [10]. They include three cytoplasmic proteins (YscL, N and Q), five IM proteins (YscR, S, T, U and V) and the OM secretin (YscC), which is absent from flagellar T3S systems.

The membrane-spanning portions of flagellar and translocation-associated T3S systems are associated with extracellular appendages including the flagellar hook and filament as well as the pilus (in plant pathogenic bacteria; up to 2 µm long) or needle (in animal pathogenic bacteria; 40-80 nm long) of translocation-associated T3S systems [11]. The pilus and needle are essential for T3S and presumably provide protein transport channels to the host-pathogen interface. They are directly or indirectly connected to the T3S translocon, which is a predicted oligomeric protein channel in the host plasma membrane and mediates effector protein translocation into the host cell [12]-[14].

One of the model systems to study T3S is the Gram-negative bacterium *Xanthomonas campestris* pv. *vesicatoria*, which causes bacterial spot disease of pepper and tomato plants. The T3S system from *X. campestris* pv. *vesicatoria* translocates approximately 30 to 40 effector proteins into the plant cell where they interfere with host cellular processes such as gene expression, signal transduction cascades and the suppression of host defense responses to the benefit of the pathogen [15]. Effector protein translocation is activated *in planta* by a yet unknown signal and depends on the chromosomal *hrp* (hypersensitive response and pathogenicity) gene cluster, which encodes the components of the T3S system [15],[16]. Mutant studies with individual *hrp* genes revealed that efficient T3S does not only depend on predicted components of the T3S system but also on control proteins – designated Hpa (Hrp associated) - that presumably regulate T3S substrate specificity and recognition. Among the control proteins is the general T3S chaperone HpaB which binds to and promotes the efficient secretion and translocation of multiple effector proteins [17]-[19]. HpaB presumably targets effector proteins to the ATPase HrcN of the T3S system which can dissociate HpaB-effector protein complexes and thus might facilitate the entry of effector proteins into the inner channel of the T3S system [20].

In addition to HpaB, the efficient translocation of effector proteins depends on HpaC, which is a T3S substrate specificity switch (T3S4) protein. HpaC promotes the secretion of translocon and effector proteins but suppresses the efficient secretion of HrpB2, which is required for T3S pilus formation [21]-[23]. Given the architecture of the T3S system, pilus assembly likely occurs prior to the secretion of translocon and effector proteins, suggesting that the substrate specificity of the T3S system switches from “early“ to “late“ substrates [14], [24], [25]. The switch is mediated by T3S4 proteins that interact with the cytoplasmic domains of members of the YscU family of IM proteins. It was proposed that T3S4 proteins induce a conformational change in the cytoplasmic domains of YscU family members that leads to an alteration in substrate recognition [3], [14], [24]. In agreement with this model, HpaC interacts with the C-terminal domain of the YscU homolog HrcU (HrcU_C_). Furthermore, the *hpaC* mutant phenotype can be suppressed by a point mutation in HrcU_C_ that likely mimicks the predicted conformational change [21], [26]. HrcU_C_ interacts with HrpB2, suggesting that it provides a docking site for early T3S substrates. However, an interaction between HrcU_C_ and late T3S substrates has not yet been observed [21]. It is therefore still unclear how late substrates are recognized by the T3S system.

In the present study, we analyzed a possible contribution of the YscQ homolog HrcQ to T3S and substrate docking. HrcQ belongs to the family of putative cytoplasmic (C) ring components of the T3S system that are proposed to form a cup-like structure with a diameter of approximately 40 nm. The predicted C ring of translocation-associated T3S systems has not yet been visualized because it presumably easily disconnects from the membrane-spanning secretion apparatus during the purification procedure. However, the C ring was visualized by electron microscopy of isolated flagellar T3S systems [27], [28]. Flagellar C rings consist of three proteins (FliG, M and N) that connect the C ring to the IM components of the T3S system such as the ATPase complex or the ring components in the IM [4], [27]-[31]. FliM and FliN share amino acid sequence similarities with predicted C ring components of translocation-associated T3S systems. Given the finding that YscQ and homologous proteins from animal pathogenic bacteria interact with effector proteins or effector-chaperone complexes, the predicted C ring of translocation-associated T3S systems was proposed to act as a recruitment platform for secreted proteins [32], [33]. We therefore investigated the contribution of HrcQ from *X. campestris* pv. *vesicatoria* to T3S and substrate binding. Mutant and protein studies revealed that HrcQ is an essential component of the T3S system and associates with the bacterial membranes under T3S-permissive conditions. *In vitro* pull-down assays showed that HrcQ interacts with conserved components of the T3S system at the IM including the IM proteins HrcV and HrcU, the ATPase HrcN and its predicted regulator HrcL. Furthermore, HrcQ binds to early and late T3S substrates and therefore is likely involved in T3S substrate recognition. Interestingly, the analysis of derivatives of the effector protein AvrBs3 suggests that the N-terminal T3S and translocation signal is dispensable for the interaction of AvrBs3 with HrcQ.

## Materials and Methods

### Bacterial Strains and Growth Conditions

Bacterial strains and plasmids used in this study are listed in Table 1. *Escherichia coli* cells were grown at 37°C in lysogeny broth (LB) or Super medium (Qiagen, Hilden, Germany). *X. campestris* pv. *vesicatoria* strains were cultivated at 30°C in nutrient-yeast-glycerol (NYG) medium [47] or in minimal medium A [48] supplemented with sucrose (10 mM) and casamino acids (0.3%). Plasmids were introduced into *E. coli* by transformation and into *X. campestris* pv. *vesicatoria* by conjugation, using pRK2013 as a helper plasmid in triparental matings [49]. Antibiotics were added to the media at the following final concentrations: ampicillin, 100 µg/ml; kanamycin, 25 µg/ml; rifampicin, 100 µg/ml; spectinomycin, 100 µg/ml; gentamycin, 7.5 µg/ml.

**Table 1:** Bacterial strains and plasmids used in this study.

	Relevant characteristics^a^			Reference or source	
***X. campestris*** ** pv. ** ***vesicatoria***					
85-10	Pepper-race 2; wild type; Rif^r^			[51], [73]	
85-10Δ*hrcQ*	*hrcQ* deletion mutant of strain 85-10			This study	
85-10Δ*hrcQ*::*hrcQ*	Derivative of strain 85-10Δ*hrcQ* carrying *hrcQ-c-myc* under control of the native promoter inserted into the *hpaFG* region			This study	
85*	85-10 derivative containing the hrpG* mutation			[34]	
85*Δ*hrcQ*	*hrcQ* deletion mutant of strain 85*			This study	
85*Δ*hrcQ*::*hrcQ*	Derivative of strain 85*Δ*hrcQ* carrying *hrcQ-c-myc* under control of the native promoter inserted into the *hpaFG* region			This study	
***E. coli***					
BL21 (DE3)	F^-^ *omp*T *hsd*S_B_ (r_B_^-^ m_B_^-^) *gal dcm* (DE3)			Stratagene, Heidelberg, Germany	
Top10	F- *mcr*A Δ(*mrr*-*hsd*RMS-*mcr*BC) Φ80*lac*ZΔM15 Δ*lac*X74 *rec*A1 *ara*D139 Δ(*araleu*) 7697 *gal*U *gal*K *rps*L (Str^R^) *end*A1 *nup*G			Invitrogen, Karlsruhe, Germany	
DH5α	F^-^ *recA hsdR17(r_k_^-^,m_k_^+^) Φ80dlacZ* Δ*M15*			Bethesda Research Laboratories, Bethesda, Md.	
DH5α λpir	F^-^ *recA hsdR17(r_k_^-^,m_k_^+^) Φ80dlacZ* Δ*M15* [λ*pir*]α			[74]	
**Plasmids**					
pBlueskript(II) KS	Phagemid, pUC derivative; Ap^r^			Stratagene	
pBRM	Golden Gate-compatible derivative of pBBR1MCS-5 for *lac*promoter-driven gene expression; contains a 3× c-Mycepitope-encoding sequence			[75]	
pBRM-P	Derivative of pBRM without *lac* promoter			This study	
pBRMhrcN	pBRM derivative encoding HrcN-c-Myc			[75]	
pBRMhrcQ	pBRM derivative encoding HrcQ-c-Myc			This study	
pBRMhrcQ_Stop_	pBRM derivative encoding HrcQ			This study	
pBRM-PhrcQ	pBRM-P derivative containing *hrcQ-c-myc* and 299 bp upstream region			This study	
pBRM-PhrcQ_Stop_	pBRM-P derivative containing *hrcQ* and 299 bp upstream region			This study	
pBRMhrcV	pBRM derivative encoding HrcV-c-Myc			N. Hartmann and D. Büttner, unpublished	
pBRMhrcV_324-645_	pBRM derivative encoding HrcV_324-645_-c-Myc			N. Hartmann and D. Büttner, unpublished	
pBRMxopJ	pBRM derivative encoding XopJ-c-Myc			[26]	
pDGW4MhpaA	Derivative of pDGW4M encoding HpaA-c-Myc			[80]	
pDMhrcL	Derivative of pDSK602 encoding HrcL-c-Myc			[20]	
pGEX-2TKM	GST expression vector; p*_tac_* GST *lacI* ^q^ pBR322 *ori;* Ap^r^, derivative ofpGEX-2TK with polylinker of pDSK604			Stratagene[38]	
pGhpaA	pGEX-2TKM derivative encoding GST-HpaA			[76]	
pGhpaB	pGEX-2TKM derivative encoding GST-HpaB			[18]	
pGhpaC	pGEX-2TKM derivative encoding GST-HpaC			[18]	
pGhrpB2	pGEX-2TKM derivative encoding GST-HrpB2			[62]	
pGhrpE	pGEX-2TKM derivative encoding GST-HrpE			[55]	
pGhrcL	pGEX-2TKM derivative encoding GST-HrcL			[20]	
pGhrcN	pGEX-2TKM derivative encoding GST-HrcN			[76]	
pGhrcQ	pGEX-2TKM derivative encoding GST-HrcQ			This study	
pGhrcU	pGEX-2TKM derivative encoding GST-HrcU			[21]	
pGhrcU_255-357_	pGEX-2TKM derivative encoding GST-HrcU_255-357_			[21]	
pGxopA	pGEX-2TKM derivative encoding GST-XopA			[17]	
pGxopJ	pGEX-2TKM derivative encoding GST-XopJ			This study	
pGxopF1	pGEX-2TKM derivative encoding GST-XopF1			[18]	
pG300	pGEX-2TKM derivative encoding GST-AvrBs3			[77]	
pG356F	pGEX-2TKM derivative encoding GST-AvrBs3Δ2 which lacks theN-terminal 152 amino acids			[17]	
pGavrBs350	pGEX-2TKM derivative encoding GST-AvrBs3_1-50_			[17]	
pLAND-P	Derivative of pOK1 carrying fragments of the *hpaFG* region flanking *lacZ*, the *lac* promoter and a triple c-Myc epitope-encoding sequence			This study	
pLAND-PhrcQ	Derivative of pLAND-P carrying *hrcQ* and 299 bp upstream region			This study	
pOK1	Suicide vector; *sacB sacQ mobRK2* oriR6K; Sm^r^			[78]	
pOKΔhrcQ	Derivative of pOK1 carrying the flanking regions of *hrcQ*			This study	
pRK2013	ColE1 replicon, TraRK^+^ Mob^+^; Km^r^			[49]	
pUC119	ColE1 replicon; Ap^r^			[79]	

a Ap, ampicillin; Km, kanamycin; Rif, rifampicin; Sm, spectinomycin; Gm, gentamycin; r, resistant.

### Plant Material and Plant Inoculations

The near-isogenic pepper cultivars Early Cal Wonder (ECW) and ECW-10R [50], [51] were grown as described previously [52]. *X. campestris* pv. *vesicatoria* strains were inoculated with a needle-less syringe into the intercellular spaces of leaves at concentrations of 2×10^8^ colony-forming units (CFU) ml^-1^ in 1 mM MgCl_2_ if not stated otherwise. The appearance of disease symptoms and the HR were scored over a period of one to nine days post inoculation (dpi). For the better visualization of the HR, leaves were bleached in 70% ethanol. Experiments were repeated at least two times. For *in planta* growth curves, bacteria were inoculated at a density of 10^4^ CFU/ml into leaves of susceptible ECW plants. Bacterial counts were determined over a period of 11 dpi as described [52].

### Generation of Expression Plasmids

For the generation of *hrcQ* expression constructs, *hrcQ* with or without stop codon was amplified by PCR from *X. campestris* pv. *vesicatoria* strain 85-10 and cloned into the Golden Gate-compatible expression vector pBRM using the type IIs restriction enzyme *Bsa*I in a single restriction/ligation reaction [53]. The broad host-range vector pBRM is a derivative of plasmid pBBR-MCS5 and allows the expression of genes under control of the *lac* promoter in frame with a 3× c-Myc epitope-encoding sequence. Alternatively, we cloned *hrcQ* including its native promoter (299 bp upstream of the translation initiation codon GTG) into plasmid pBRM-P, which is a derivative of pBRM that lacks the *lac* promoter (Table 1). To obtain GST fusion constructs, *hrcQ* and *xopJ* were amplified by PCR from *X. campestris* pv. *vesicatoria* strain 85-10 and cloned into the *Eco*RI/*Xho*I sites of vector pGEX, in frame with a GST-encoding sequence. Primer sequences are available upon request.

### Construction of *X. campestris* pv. *vesicatoria* Deletion Mutants

For the generation of a genomic *hrcQ* deletion mutant, 700-bp and 750-bp regions flanking *hrcQ* and spanning part of the 5′- and 3′- region of *hrcQ* were amplified by PCR and cloned into the *Bam*HI and *Apa*I sites of plasmid pOK1. The resulting construct pOKΔhrcQ was conjugated into strains 85-10 and 85*. Double cross-overs resulted in strains 85-10Δ*hrcQ* and 85*Δ*hrcQ* that were selected as described previously [44] and contain a deletion of codons 11 to 243 of *hrcQ* followed by a nonsense mutation.

### Construction of the Suicide Vector pLAND-P

To generate plasmid pLAND-P, we amplified 850- and 770-bp fragments spanning *hpaF* and the 5′-region of *hpaG* as well as the 3′-region of *hpaG*, respectively, by primers hpaF-for (5′-TTTGGTCTCT*CATG*CATGCGGCGATGGCAGTC-3′, hpaF-rev (5′-TTTGGTCTCT*AGAC*CCCATGGCAGCGAGAGGTTGCGAAG), hpaG-for (5′-TTTGGTCTCT*GTCT*CTAATTATCGTTGAGCTGAGCAG-3′) and hpaG-rev (5′-TTTGGTCTCT*GATC* CTCCTGCGTGTGCATG-3′). PCR amplicons were digested with *Bsa*I (restriction sites are underligned in primer sequences, overhangs are written in italics) and ligated into the *Bam*HI and *Nco*I sites of the suicide vector pOK1, thus generating pOKhpaFG. The ligation reaction led to a mutation in the internal *Nco*I site of pOK1 and to the generation of a *Bsa*I and *Nco*I site between both ligated amplicons. Next, we amplified the *lacZ* gene including the *lac* promoter and the 3× c-Myc epitope-encoding sequence from plasmid pBRM-P using primers lacZ-P-for (5′-TTTCGTCTCTA*ATTC*AGAGACCGCAGCTGGCACGACAG-3′) and Myc-rev (5′-TTTCGTCTCT*CATG*GTCAGTTCAAGTCTTCTTC-3′). The amplicon was digested with *Esp*3I and ligated into the *Bsa*I/*Nco*I-digested pOKhpaFG, resulting in pLAND-P. The introduction of a *Bsa*I site by primer lacZ-P-for and the presence of a *Bsa*I site upstream of the 3× c-Myc epitope-encoding sequence allows the targeted ligation of genes of interest in the opposite orientation of the *hpaFG* transcription. To generate pLAND-PhrcQ, *hrcQ* including its native promoter was amplified and cloned into pLAND-P in a single restriction/ligation reaction using *Bsa*I and ligase.

### Glutathione S-transferase (GST) Pull-down Assays

For GST pull-down assays, GST and GST fusion proteins were synthesized in *E. coli* BL21(DE3). Bacterial cells from 50 ml cultures were resuspended in phosphate-buffered saline (PBS) and broken with a French press. Insoluble cell debris were removed by centrifugation, and soluble GST and GST fusion proteins were immobilized on a glutathione sepharose matrix according to the manufacturer’s instructions (GE Healthcare, Munich, Germany). Unbound proteins were removed by washing twice with PBS, and the glutathione sepharose matrix was incubated with 600 µl *E. coli* cell lysates containing the putative interaction partners for 1 to 2 h at 4°C. Unbound proteins were removed by washing four times with PBS and bound proteins were eluted with 10 mM reduced glutathione at room temperature for 2 h. 10 µl total protein lysates and 20 µl eluted proteins were analyzed by SDS-PAGE and immunoblotting using antibodies specific for the c-Myc epitope and GST, respectively (Roche Applied Science, Mannheim, Germany; GE Healthcare) [22]. Horseradish peroxidase-labeled anti-mouse and anti-goat antibodies (GE Healthcare) were used as secondary antibodies. Antibody reactions were visualized by enhanced chemiluminescence (GE Healthcare).

### Generation of Polyclonal HrcQ Antibodies

For the generation of HrcQ antibodies, rabbits were immunized with a HrcQ-specific peptide (AEVIAFERDAEPDD, amino acids 82 to 95) (Biogenes, Berlin, Germany). The serum after the second booster injection was used for immunoblot analysis.

### Protein Secretion Studies and Immunoblot Analysis

T3S assays were performed as described previously [54]. Briefly, bacteria were incubated in secretion medium and equal amounts of bacterial total cell extracts and culture supernatants were analyzed by SDS-PAGE and immunoblotting. We used polyclonal antibodies specific for HrpF [55], HrpB2 [22] and HrcQ (this study), and monoclonal anti-c-Myc (Roche Applied Science, Mannheim, Germany) and anti-GST antibodies (Amersham Pharmacia Biotech, Freiburg, Germany). Horseradish peroxidase-labeled anti-rabbit antibodies (GE Healthcare) were used as secondary antibodies. Experiments were repeated at least two times. Blots were routinely reacted with an antibody specific for the intracellular proteins HrcN or HrcJ to ensure that no bacterial lysis had occurred [22] (data not shown).

### Subcellular Localisation Studies

For subcellular localization of proteins, bacteria were grown overnight in minimal medium A supplemented with sucrose and casamino acids. Bacterial cells from 50 ml cultures were resuspended in 3 ml 100 mM HEPES (4-(2-hydroxyethyl)-1-piperazineethanesulfonic acid) and lysed with a French press. Cell debris were removed by centrifugation and 1 ml of each lysate was centrifuged at 200.000 g for 90 minutes at 4°C. Similar protein levels were adjusted according to OD_600_. The pellet, which corresponds to the membrane fraction, was resuspended in 1 ml 100 mM HEPES. Membrane and soluble fractions were mixed with Laemmli buffer and 20 µl total bacterial lysate, membrane and soluble fractions were analyzed by SDS-PAGE, Coomassie staining (data not shown) and immunoblotting.

## Results

### HrcQ from *X. campestris* pv. *vesicatoria* is Essential for Pathogenicity and T3S

*hrcQ* from *X. campestris* pv. *vesicatoria* strain 85-10 is the first gene of the *hrpD* operon of the *hrp* gene cluster and encodes a predicted protein of 304 amino acids that is conserved in *Xanthomonas* spp. (91 to 93% amino acid identity with HrcQ proteins from *Xanthomonas oryzae* pv. *oryzae*, *X. oryzae* pv. *oryzicola*, *Xanthomonas axonopodis* pv. *citri* and *X. axonopodis* pv. *glycines*; 76% amino acid identity with HrcQ from *X. campestris* pv. *campestris*). The C-terminal portion of HrcQ shares weak sequence similarity (27 to 33% amino acid sequence identity) with members of the YscQ protein family from animal pathogenic bacteria. To study the contribution of HrcQ to pathogenicity of *X. campestris* pv. *vesicatoria*, we generated a *hrcQ* deletion mutant carrying an in-frame deletion of codons 11 to 243 followed by a nonsense mutation. The *hrcQ* deletion was introduced into the genome of the wild-type strain 85-10 and its derivative 85-10hrpG* (85*), which contains HrpG*, a constitutively active version of the key regulatory protein HrpG. HrpG* activates – in most cases via the transcriptional regulator HrpX – the expression of a genome-wide regulon including *hrp*, putative virulence and effector genes [19], [34]-[36].

**Figure 1:**
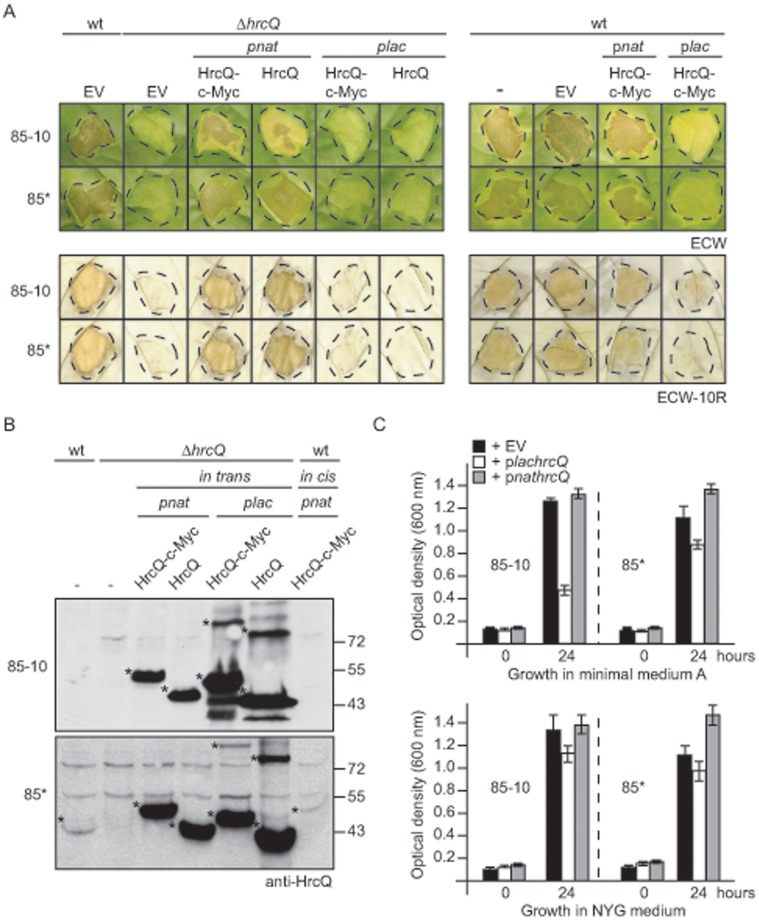
HrcQ contributes to bacterial pathogenicity and *in planta* growth. (A) Ectopic expression of *hrcQ* under control of the native promoter partially restores pathogenicity of *hrcQ* deletion mutant strains. *X. campestris* pv. *vesicatoria* strains 85-10 (wt), 85-10Δ*hrcQ* (Δ*hrcQ*), 85* (wt) and 85*Δ*hrcQ* (Δ*hrcQ*) without expression construct (-) or carrying plasmid pBRM (EV) or derivatives thereof expressing *hrcQ* or *hrcQ-c-myc* under control of the native (p*nat*) or the *lac* (p*lac*) promoter as indicated were inoculated into leaves of susceptible ECW and resistant ECW-10R pepper plants. Bacterial strains in the left panel (complementation studies) were inoculated at a density of 10^8^ CFU ml^-1^, strains in the right panel (analysis of dominant-negative effects) at a density of 2×10^8^ CFU ml^-8^. Disease symptoms were photographed 8 dpi. For the better visualization of the HR, leaves were bleached in ethanol 2 dpi. Dashed lines mark the infiltrated areas. (B) Immunodetection of HrcQ and HrcQ-c-Myc. *X. campestris* pv. *vesicatoria* strains 85-10 (wt), 85-10Δ*hrcQ* (Δ*hrcQ*), 85* (wt) and 85*Δ*hrcQ* (Δ*hrcQ*) without expression construct (-) or encoding HrcQ or HrcQ-c-Myc *in trans* or *in cis* under control of the native (p*nat*) or the *lac* (p*lac*) promoter as indicated were grown in minimal medium A. Equal amounts of total cell extracts were analyzed by immunoblotting, using HrcQ-specific antibodies. HrcQ-specific signals are marked with asterisks, additional signals result from unspecific binding of the antibodies. The HrcQ-specific signals above 72 kDa presumably correspond to HrcQ protein complexes that were not dissociated by SDS-PAGE. (C) Overexpression of *hrcQ* leads to reduced bacterial *in vitro* growth. *X. campestris* pv. *vesicatoria* strains 85-10 and 85* carrying plasmid pBRM (EV) or expressing *hrcQ-c-myc* under control of the *lac* (p*lachrcQ*) or the native (p*nathrcQ*) promoter were grown over night in complex NYG medium and resuspended in minimal medium A or NYG medium at an optical density (OD_600 nm_) of 0.2. The cultures were incubated at 30°C and the optical density was measured over a period of 24 h. Error bars represent standard deviations.

For infection studies, *hrcQ* wild-type and deletion mutant strains were inoculated into leaves of susceptible ECW and resistant ECW-10R pepper plants. ECW-10R plants carry the *Bs1* resistance gene and induce the hypersensitive response (HR) upon recognition of the type III effector AvrBs1, which is delivered by strain 85-10 [37], [38]. The HR is a rapid local cell death at the infection site that restricts bacterial ingress and is activated upon detection of individual bacterial effector proteins (also designated Avr [avirulence] proteins) by the plant surveillance system [39]. As expected, strains 85-10 and 85* induced disease symptoms in ECW and the HR in ECW-10R pepper plants whereas no reactions were observed for the respective *hrcQ* deletion mutants (Fig. 1A). Ectopic expression of HrcQ-c-Myc or untagged HrcQ derivatives under control of the native promoter (see Materials and Methods) complemented the mutant phenotype of strain 85*Δ*hrcQ* with respect to HR induction and disease symptoms when the bacteria were inoculated at densities of 10^8 ^CFU/ml. At lower inoculation densities, however, expression of *hrcQ* from the native promoter only partially restored symptom formation (Fig. S1). This was also observed in strain 85-10Δ*hrcQ*, even with higher inoculation densities (Fig. 1A; data not shown). To confirm that HrcQ derivatives were stably synthesized, protein extracts of bacterial cells grown in minimal medium were analyzed by immunoblotting, using HrcQ-specific antibodies. As expected, *lac* promoter-driven expression of *hrcQ* and *hrcQ-c-myc* led to increased protein amounts when compared with *hrcQ* derivatives expressed under control of the native promoter in strain 85-10Δ*hrcQ*. The amounts of the native HrcQ protein were significantly lower and were only detectable in protein extracts of strain 85* which constitutively expresses the *hrp* genes (Fig. 1B).

We also introduced an expression construct encoding a C-terminally c-Myc epitope-tagged derivative of the ATPase HrcN under control of the *lac* promoter into strains 85-10Δ*hrcQ* and 85*Δ*hrcQ.* It was previously shown that the phenotype of flagellar C ring mutants can be partially suppressed upon ectopic expression of the ATPase-encoding gene *fliI* or upon upregulation of the regulatory genes *flhDC* [40], [41]. Notably, however, expression of *hrcN-c-myc* did not alter the *hrcQ* deletion mutant phenotype (Fig. S2). This was probably not caused by a potential negative effect of elevated HrcN levels because we have previously shown that overexpression of *hrcN* in strain 85-10 does not alter the wild-type *in planta* phenotype [20]. We therefore conclude that the loss of *hrcQ* cannot be counteracted by elevated levels of the T3S-ATPase.

### Overexpression of *hrcQ* Exerts a Dominant-negative Effect on Pathogenicity and *in vitro* Bacterial Growth

Given the lack of complementation of the *hrcQ* mutant phenotype by *lac* promoter-driven expression of *hrcQ*, we analyzed whether the overexpression of *hrcQ* is detrimental for pathogenicity. For this, the wild-type strain 85-10 carrying plasmid pBRM or *hrcQ* expression constructs was inoculated into susceptible and resistant pepper plants. Fig. 1A shows that the expression of *hrcQ-c-myc* under control of the native promoter did not significantly interfere with pathogenicity of strain 85-10. By contrast, the *lac* promoter-driven expression of *hrcQ-c-myc* led to severely reduced plant reactions (Fig. 1A). Similar phenotypes were observed with derivatives of strain 85*, suggesting that the dominant-negative effect of *hrcQ* overexpression could not be counteracted by the constitutive expression of T3S genes (Fig. 1A).

To investigate whether the ectopic expression of *hrcQ* exerts a general negative effect on bacterial fitness, we also analyzed bacterial growth *in vitro.* When strains 85-10 and 85* carrying plasmid pBRM or *hrcQ* expression constructs were grown in minimal medium, the overexpression of *hrcQ* from the *lac* promoter led to a significant reduction of bacterial growth (Fig. 1C). This was also observed when bacteria were grown in complex medium NYG. However, the negative effect of *hrcQ* overexpression was less pronounced in NYG than in minimal medium, suggesting that the interference of increased HrcQ levels with bacterial fitness also depends on the culture medium (Fig. 1C).

### HrcQ is Essential for T3S *in vitro*

**Figure 2:**
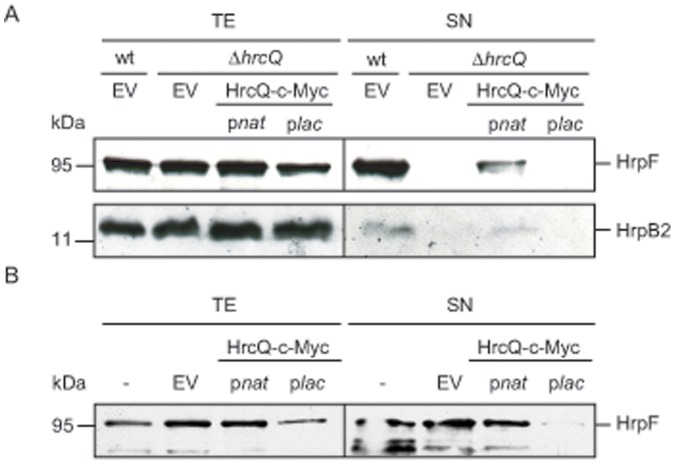
HrcQ is essential for T3S of the translocon protein HrpF and the early T3S substrate HrpB2. (A) Strains 85* (wt) and 85*Δ*hrcQ* (Δ*hrcQ*) carrying plasmid pBRM (EV) or expression constructs encoding HrcQ-c-Myc under control of the native (*pnat*) or the *lac* (*plac*) promoter were incubated in secretion medium. Total cell extracts (TE) and culture supernatants (SN) were analyzed by immunoblotting using antibodies specific for HrpF and HrpB2, respectively. (B) *lac* promoter-driven expression of *hrcQ-c-myc* exerts a negative effect on T3S of HrpF. Strain 85* without expression construct (-), with plasmid pBRM (EV) or derivatives thereof encoding HrcQ-c-Myc as described in panel A were incubated in secretion medium. TE and SN were analyzed by immunoblotting using HrpF-specific antibodies.

To investigate a potential influence of HrcQ on T3S *in vitro*, strains 85* and 85*Δ*hrcQ* were incubated in secretion medium. Total cell extracts and culture supernatants were analyzed by immunoblotting, using specific polyclonal antisera against the translocon protein HrpF and the early T3S substrate HrpB2, respectively. As expected, HrpF and HrpB2 were secreted by strain 85* but were not detectable in the supernatant of strain 85*Δ*hrcQ*, suggesting that HrcQ is essential for T3S (Fig. 2A). The secretion deficiency was partially complemented by ectopic expression of *hrcQ-c-myc* under control of the native but not of the *lac* promoter (Fig. 2A).

To test a possible negative effect of *hrcQ* overexpression on T3S, we performed additional secretion assays with strain 85* carrying pBRM or *hrcQ* expression constructs. The analysis of culture supernatants by immunoblotting revealed that the *lac* promoter-driven expression of *hrcQ-c-myc* in strain 85* interfered with the efficient secretion of the translocon protein HrpF (Fig. 2B). By contrast, expression of *hrcQ-c-myc* under control of the native promoter did not significantly affect HrpF secretion (Fig. 2B). This is in agreement with the results of the infection experiments (see above) and suggests that the *lac* promoter-driven expression of *hrcQ* derivatives exerts a negative effect on T3S and pathogenicity.

### Insertion of *hrcQ* into a Genomic "Landing Platform" Fully Restores Pathogenicity of the *hrcQ* Deletion Mutant

**Figure 3:**
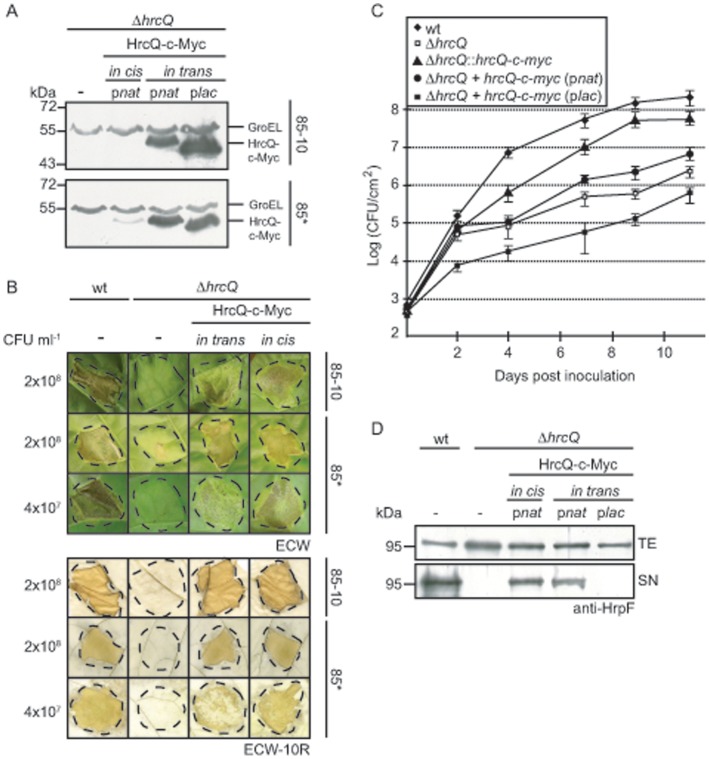
Complementation studies with chromosomally encoded *hrcQ-c-myc.* (A) Immunodetection of *in cis-*encoded HrcQ-c-Myc. *X. campestris* pv. *vesicatoria* strains 85-10Δ*hrcQ* (Δ*hrcQ*) and 85*Δ*hrcQ* (Δ*hrcQ*) carrying plasmid pBRM (-) or encoding HrcQ-c-Myc in the chromosome (*in cis*) or on an expression plasmid (*in trans*) under control of the native (p*nat*) or the *lac* (p*lac*) promoter as indicated were grown in minimal medium A. Equal amounts of total cell extracts were analyzed by immunoblotting, using c-Myc epitope- and GroEL-specific antibodies, respectively. GroEL was analyzed as a loading control. (B) Insertion of *hrcQ-c-myc* into the *hpaFG* region restores wild-type symptom formation in *hrcQ* deletion mutants. Strains 85-10 (wt), 85-10Δ*hrcQ* (Δ*hrcQ*), 85* (wt) and 85*Δ*hrcQ* (Δ*hrcQ*) carrying plasmid pBRM (-) or encoding HrcQ-c-Myc under control of the native promoter from plasmid pBRM-P (*in trans*) or in the chromosome (*in cis*) were inoculated at bacterial cell densities of 2×10^8^ or 4×10^7^ CFU/ml as indicated into leaves of susceptible ECW and resistant ECW-10R pepper plants. Disease symptoms were photographed 9 dpi. For the better visualization of the HR, leaves were bleached in ethanol 2 dpi. Dashed lines mark the infiltrated areas. (C) *In planta* growth of a *hrcQ* deletion mutant strain can be partially restored upon expression of *hrcQ in cis* or *in trans*. *X. campestris* pv. *vesicatoria* strains 85-10 (wt), 85-10Δ*hrcQ* (Δ*hrcQ*), 85-10Δ*hrcQ*::*hrcQ-c-myc* (Δ*hrcQ*::*hrcQ-c-myc*) and 85-10Δ*hrcQ* expressing *hrcQ-c-myc* under control of the native (p*nat*) or the *lac* (p*lac*) promoter as indicated were inoculated into leaves of susceptible ECW pepper plants. Bacterial growth was analyzed over a period of 11 days. Values are the mean of three samples from three plants. Error bars represent standard deviations. The experiment was repeated two times. One representative experiment is shown. (D) *In vitro* T3S assays with *hrcQ* deletion mutants encoding HrcQ-c-Myc on the chromosome or on expression plasmids. Strains 85* (wt) and 85*Δ*hrcQ* (Δ*hrcQ*) carrying plasmid pBRM (-) or encoding HrcQ-c-Myc in the chromosome (*in cis*) or on an expression plasmid (*in trans*) under control of the native (p*nat*) or the *lac* (p*lac*) promoter as indicated were incubated in secretion medium. Total cell extracts (TE) and culture supernatants (SN) were analyzed by immunoblotting using HrpF-specific antibodies.

The negative effect of increased HrcQ levels prompted us to perform additional complementation studies with *hrcQ* deletion strains in which *hrcQ* was expressed *in cis* under control of the native promoter. For this, *hrcQ* was inserted into the genomic *hpaF-hpaG* locus in the flanking region of the *hrp* gene cluster by homologous recombination. *hpaF* and *hpaG* encode proteins with homology to the N- and C-terminal regions, respectively, of the leucine-rich repeat-containing effector protein XopAE from *Xanthomonas* spp. It was previously shown that mutations in the *hpaFG* region do not significantly interfere with bacterial pathogenicity [42], [43]. For the insertion of *hrcQ* into the *hpaFG* region of *X. campestris* pv. *vesicatoria*, we generated a novel Golden Gate-compatible suicide vector (pLAND-P; see Materials and Methods) that contains fragments of *hpaF* and *hpaG* flanking the *lacZ* gene and a triple c-Myc epitope-encoding sequence. Two recognition sites for the type IIs restriction enzyme *Bsa*I upstream and downstream of *lacZ* allowed the targeted insertion of genes by a Golden Gate reaction (see Materials and Methods) in the opposite orientation to the direction of the *hpaFG* transcription which excludes that their expression is controlled by the *hpaFG* promoter (Fig. S3). *hrcQ* including its native promoter was cloned into pLAND-P and inserted into the *hpaFG* region of strains 85-10Δ*hrcQ* and 85*Δ*hrcQ*, respectively, by homologous recombination as was described previously [44]. Immunoblot analysis of bacterial protein extracts showed that chromosomal *hrcQ-c-myc* was expressed in strain 85*Δ*hrcQ::hrcQ-c-myc* and that the amounts of HrcQ-c-Myc were significantly reduced when compared with strain 85*Δ*hrcQ* carrying *hrcQ-c-myc* expression plasmids (Fig. 1B and 3A). As expected, chromosomally encoded HrcQ-c-Myc was not detectable in protein extracts of strain 85-10Δ*hrcQ::hrcQ-c-myc* which contains the wild-type *hrpG* gene and therefore does not efficiently express the *hrp* genes when bacteria are cultivated in non-inducing minimal medium (Fig. 1B and 3A).

Plant infection experiments revealed that the *in cis* expression of *hrcQ-c-myc* fully complemented the *hrcQ* mutant phenotype of both strains 85-10Δ*hrcQ* and 85*Δ*hrcQ*, even at low inoculation densities (Fig. 3B). We also studied *in planta* growth of strain 85-10Δ*hrcQ* carrying different *hrcQ* expression constructs over a period of eleven days. Fig. 3C shows that the wild-type strain 85-10 grew to a density of more than 10^8^ CFU/cm^2^ of the infected plant tissue, whereas growth of the *hrcQ* deletion mutant was significantly reduced. Expression of *hrcQ* under control of the *lac* promoter led to a further reduction of *in planta* growth, suggesting that *hrcQ* overexpression has a dominant-negative effect on *in planta* growth (Fig. 3C). Bacterial growth of strain 85-10Δ*hrcQ* was slightly increased upon ectopic expression of *hrcQ* under control of the native promoter. By contrast, a more significant increase of bacterial *in planta* multiplication was observed when *hrcQ-c-myc* was introduced into the genome of strain 85-10Δ*hrcQ*, however, wild-type bacterial growth levels were not restored (Fig. 3C). We conclude that the *in cis* expression of *hrcQ* under control of the native promoter complements the phenotype of the *hrcQ* deletion mutant with respect to plant reactions and partially complements the defect in *in planta* multiplication. A partial complementation was also observed for the *in vitro* T3S of the translocon protein HrpF (Fig. 3D).

### HrcQ Localizes to the Bacterial Membranes upon Activation of the T3S System

**Figure 4:**
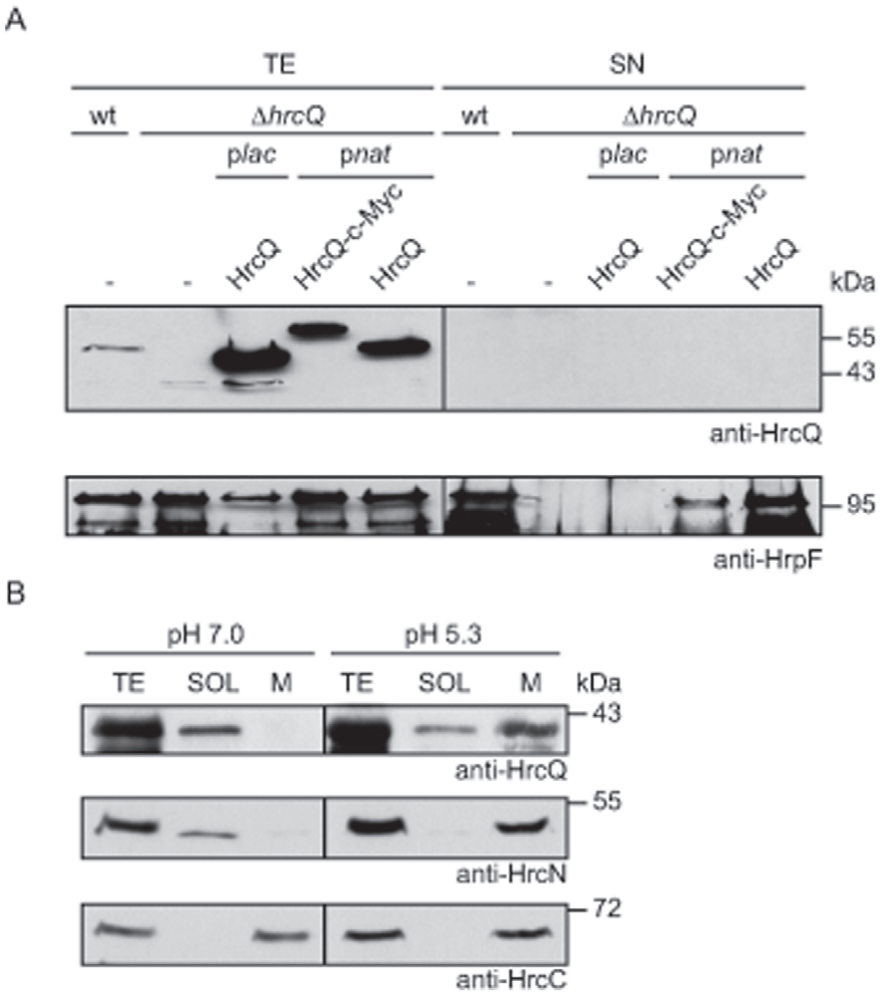
Secretion and subcellular fractionation studies with HrcQ. (A) HrcQ is not detectable in the culture supernatant under T3S-inducing conditions. Strains 85* (wt) and 85*Δ*hrcQ* (Δ*hrcQ*) carrying plasmid pBRM (-) or expressing *hrcQ* or *hrcQ-c-myc* under control of the native (p*nat*) or the *lac* (p*lac*) promoter as indicated were incubated in secretion medium. Total cell extracts (TE) and culture supernatants (SN) were analyzed by immunoblotting, using HrcQ- and HrpF-specific antibodies. HrpF was analyzed as a positive control for T3S. (B) HrcQ preferentially associates with the bacterial membranes under T3S-permissive conditions. Strain 85* was grown in minimal medium A supplemented with sucrose and casamino acids under secretion-permissive (pH 5.3) and non-permissive (pH 7.0) conditions. Membrane (M) and soluble (SOL) fractions were separated by ultracentrifugation and analyzed by immunoblotting, using antibodies directed against HrcQ, HrcN and HrcC, respectively.

Given the homology of HrcQ to predicted C ring components, we investigated the localization of HrcQ in *X. campestris* pv. *vesicatoria*. For this, we first performed secretion studies with strains 85* and 85*Δ*hrcQ* carrying *hrcQ* or *hrcQ-c-myc* expression constructs. Fig. 4A shows that HrcQ and derivatives were present in the total cell extracts but not detectable in the culture supernatants when bacteria were incubated in secretion medium, suggesting that HrcQ is not secreted by the T3S system. Next, we separated membrane fractions and soluble proteins of strain 85* by ultracentrifugation after cultivation of the bacteria in miminal medium A at pH 7.0 (nonpermissive conditions for T3S) or pH 5.3 (T3S-permissive conditions). At pH 7.0, HrcQ was predominantly present in the soluble fraction. By contrast, at pH 5.3 the amounts of HrcQ in the membrane fraction were significantly increased, suggesting a membrane association of HrcQ upon activation of the T3S system (Fig. 4B). When the blots were reprobed with antibodies specific for the ATPase HrcN and the OM secretin HrcC, HrcC was mainly present in the membrane fraction under both secretion-permissive and nonpermissive conditions. By contrast, the ATPase HrcN was detected in the soluble fraction at pH 7.0 whereas the amounts of HrcN in the membrane fraction were significantly increased under T3S-permissive conditions as was reported previously [20] (Fig. 4B).

### HrcQ Interacts with itself and with IM-associated Components of the T3S System

**Figure 5:**
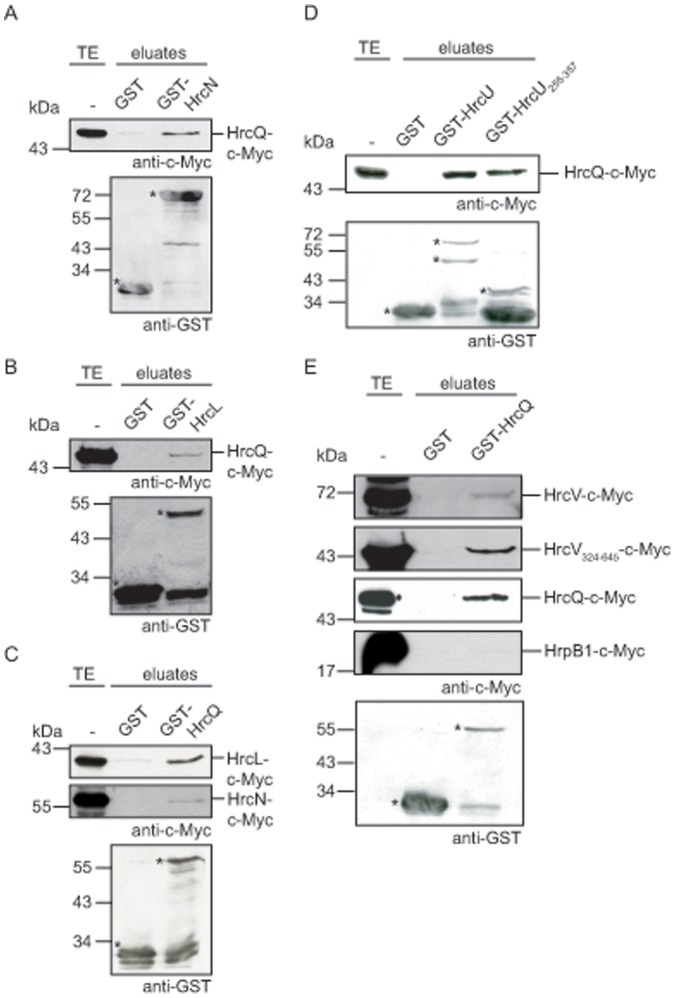
HrcQ interacts with itself, HrcN, HrcL and the cytoplasmic domains of HrcU and HrcV. (A) GST pull-down assays with HrcN. GST and GST-HrcN were immobilized on glutathione sepharose and incubated with a bacterial lysate containing HrcQ-c-Myc. Total cell extracts (TE) and eluted proteins (eluates) were analyzed by immunoblotting using c-Myc epitope- and GST-specific antibodies. GST and GST fusion proteins are marked with asterisks, additional bands correspond to degradation products. (B) GST pull-down assays with HrcL. GST and GST-HrcL were immobilized on glutathione sepharose and incubated with a bacterial lysate containing HrcQ-c-Myc. TE and eluates were analyzed as is described in panel A. (C) GST-HrcQ interacts with HrcL and HrcN. GST and GST-HrcQ were immobilized on glutathione sepharose and incubated with bacterial lysates containing HrcL-c-Myc and HrcN-c-Myc, respectively. TE and eluates were analyzed as is described in panel A. One representative blot incubated with GST-specific antibodies is shown. (D) HrcQ interacts with the cytoplasmic domain of HrcU. GST, GST-HrcU and GST-HrcU_255-357_ were immobilized on glutathione sepharose and incubated with a bacterial lysate containing HrcQ-c-Myc. TE and eluates were analyzed as is described in panel A. GST-HrcU is cleaved at the conserved NPTH motif. The signals detected by the GST-specific antibody therefore correspond to GST-HrcU and the N-terminal cleavage product [26]. (E) HrcQ interacts with the cytoplasmic domain of HrcV and with itself. GST and GST-HrcQ were immobilized on glutathione sepharose and incubated with bacterial lysates containing HrcV-c-Myc, HrcV_324-645_-c-Myc, HrcQ-c-Myc or HrpB1-c-Myc. TE and eluates were analyzed as is described in panel A. One representative blot incubated with GST-specific antibodies is shown.

Given the predicted role of HrcQ as a C ring component, HrcQ is likely to interact with other cytoplasmic or IM-associated proteins of the T3S system. To test this possibility, GST fusions of the cytoplasmic T3S ATPase HrcN and its predicted regulator HrcL were immobilized on glutathione sepharose and incubated with a bacterial lysate containing HrcQ-c-Myc. Bound proteins were eluted from the matrix and analyzed by immunoblotting using c-Myc epitope- and GST-specific antibodies. HrcQ-c-Myc specifically coeluted with GST-HrcN and GST-HrcL but not with GST alone, suggesting that it interacts with the ATPase HrcN and its predicted regulator HrcL (Fig. 5A and B). Both interaction were also shown in the opposite direction with GST-HrcQ and C-terminally c-Myc epitope-tagged derivatives of HrcL and HrcN (Fig. 5C).

We also analyzed a possible interaction of HrcQ with the IM proteins HrcV and HrcU. These proteins consist of eight and four transmembrane domains, respectively, and cytoplasmic domains that might be involved in T3S substrate docking [45]. GST pull-down assays revealed that HrcQ-c-Myc coelutes with GST fusions of HrcU and the C-terminal cytoplasmic domain of HrcU, respectively, spanning amino acids 255 to 357 (Fig. 5D). This region of HrcU was previously shown to interact with the early T3S substrate HrpB2, the T3S4 protein HpaC and the ATPase HrcN [20], [21]. We also observed interactions between GST-HrcQ and C-terminally c-Myc epitope-tagged derivatives of HrcV and the HrcV deletion derivative HrcV_324-645_-c-Myc, which corresponds to the cytoplasmic domain of HrcV [45], suggesting that HrcQ interacts with the cytoplasmic domains of both HrcU and HrcV (Fig. 5E).

As HrcQ is homologous to predicted C ring components that likely form a ring complex, we also investigated a possible self-interaction of HrcQ. When analyzed by GST pull-down assays, HrcQ-c-Myc was detected in the eluate of GST-HrcQ but not of GST, suggesting that HrcQ can interact with itself (Fig. 5E). Notably, no interaction was detected between GST-HrcQ and a C-terminally c-Myc epitope-tagged derivative of HrpB1, which is a yet uncharacterized predicted component of the T3S system [22] (Fig. 5E). This suggests that the observed protein-protein interactions were not caused by unspecific protein binding to GST-HrcQ.

### HrcQ Interacts with the T3S4 Protein HpaC and T3S Substrates

**Figure 6:**
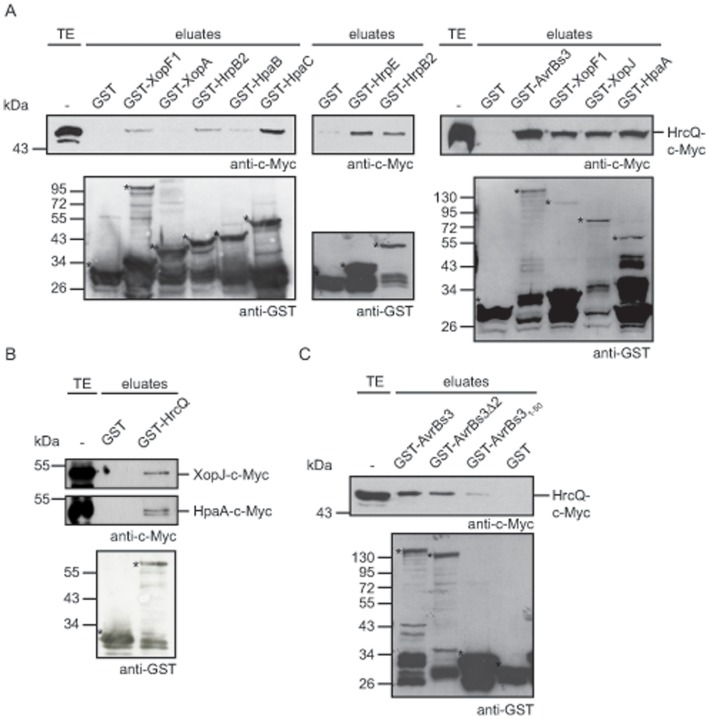
HrcQ provides a docking site for early and late T3S substrates. (A) HrcQ interacts with T3S substrates and the T3S4 protein HpaC. GST, GST-XopF1, GST-XopA, GST-HrpB2, GST-HpaB, GST-HpaC, GST-HrpE, GST-AvrBs3, GST-XopJ and GST-HpaA were immobilized on glutathione sepharose and incubated with bacterial lysates containing HrcQ-c-Myc. Total cell extracts (TE) and eluted proteins (eluates) were analyzed by immunoblotting using c-Myc epitope- and GST-specific antibodies. GST and GST fusion proteins are marked with asterisks, additional bands correspond to degradation products. (B) HrcQ interacts with XopJ and HpaA. GST and GST-HrcQ were immobilized on glutathione sepharose and incubated with bacterial lysates containing XopJ-c-Myc and HpaA-c-Myc, respectively. TE and eluates were analyzed as is described in panel A. One representative blot incubated with GST-specific antibodies is shown. (C) The N-terminal region of AvrBs3 is dispensable for the interaction with HrcQ. GST, GST-AvrBs3, GST-AvrBs3Δ2 and GST-AvrBs3_1-50_ were immobilized on glutathione sepharose and incubated with a bacterial lysate containing HrcQ-c-Myc. TE and eluates were analyzed as is described in panel A.

It was previously reported that predicted C ring components from animal pathogenic bacteria interact with effector proteins or effector-chaperone complexes [32], [33], [46]. We therefore tested a possible interaction of HrcQ with secretion substrates as well as with the general T3S chaperone HpaB and the T3S4 protein HpaC, which are probably both involved in the targeting of secreted proteins to the T3S system [17], [18], [21]. All candidate interaction partners were immobilized as GST fusion proteins and incubated with bacterial lysates containing HrcQ-c-Myc. Eluted proteins were analyzed by immunoblotting as described above. Fig. 6A shows that HrcQ-c-Myc coeluted with GST-HpaC and GST fusions of the early T3S substrate HrpB2, the pilus protein HrpE and the effector protein XopF1. By contrast, only small amounts of HrcQ-c-Myc were detected in the eluates of GST fusions of the predicted translocon protein XopA and the T3S chaperone HpaB (Fig. 6A).

The interaction between HrcQ and the effector protein XopF1 prompted us to investigate whether HrcQ can also bind to additional effector proteins. We therefore performed pull-down assays with GST fusions of the effector proteins AvrBs3, XopJ and HpaA. Notably, HrcQ-c-Myc was detected in the eluates of all GST-effector fusions but not of GST alone, suggesting that it provides a binding site for several effector proteins (Fig. 6A). We also performed the interaction studies in the opposite direction with GST-HrcQ and C-terminally c-Myc epitope-tagged derivatives of putative HrcQ interaction partners. Fig. 6B shows that the effector proteins XopJ-c-Myc and HpaA-c-Myc were detected in the eluate of GST-HrcQ but not of GST alone. By contrast, XopF1-c-Myc and HrpB2-c-Myc were not detected in the eluate of GST-HrcQ, suggesting that they did not interact with the GST-HrcQ fusion protein (data not shown). However, it cannot be excluded that the C-terminal c-Myc epitope of XopF1 and HrpB2 masks a protein region and/or affects the folding of both proteins and thus the interaction with HrcQ. Notably, we have previously observed that a C-terminally c-Myc epitope-tagged derivative of HrpB2 does not complement the *hrpB2* mutant phenotype, suggesting that the c-Myc epitope interferes with protein function [21].

### The N-terminal Region of AvrBs3 is Dispensable for the Interaction with HrcQ

As it is assumed that the targeting of T3S substrates and/or their recognition by components of the secretion apparatus often depends on a secretion signal in the N-terminal regions of secreted proteins, we wondered whether this signal is required for the binding of effector proteins to HrcQ. T3S signals of effector proteins from *X. campestris* pv. *vesicatoria* have not yet been studied in detail, but a T3S signal and HpaB-binding site were previously localized in the N-terminal 50 amino acids of the TAL effector AvrBs3 [17]. To investigate the contribution of this protein region to the interaction of AvrBs3 with HrcQ, we performed additional pull-down assays with HrcQ-c-Myc and GST fusion proteins containing the full-length AvrBs3 protein, the N-terminal 50 amino acids or an N-terminally truncated derivative of AvrBs3, designated AvrBs3Δ2, which lacks the first 152 amino acids. HrcQ-c-Myc coeluted with GST-AvrBs3 and GST-AvrBs3Δ2, suggesting that the N-terminal region of AvrBs3 is dispensable for the interaction with HrcQ. By contrast, the amounts of HrcQ-c-Myc that were detected in the eluate of GST-AvrBs3_1-50_ were significantly reduced (Fig. 6C). Thus, the N-terminal 50 amino acids of AvrBs3 are probably not sufficient for the efficient binding of HrcQ. Taken together, we conclude from our findings that HrcQ interacts with components and substrates of the T3S system and that the T3S signal of AvrBs3 is not required for the interaction with HrcQ.

## Discussion

In this study we identified HrcQ as an essential component and a potential substrate docking site of the T3S system from *X. campestris* pv. *vesicatoria*. HrcQ is homologous to predicted C ring components of T3S systems (YscQ protein family) that are assumed to form a cytoplasmic cup-like structure and might provide a binding site for chaperone-effector complexes [14]. Given that putative C ring components have mainly been studied in animal pathogenic bacteria, our data present the first detailed functional characterization of an YscQ family member from a plant pathogen. Genetic approaches revealed that HrcQ from *X. campestris* pv. *vesicatoria* is essential for T3S of early and late substrates as well as for pathogenicity and bacterial *in planta* growth. Loss of pathogenicity was also observed for *hrcQ* mutants that express hrpG* and thus encode a constitutively active version of the key regulator HrpG (Fig. 1). Furthermore, the *hrcQ* mutant phenotype could not be complemented by overexpression of the ATPase-encoding *hrcN* gene, suggesting that enhanced expression levels of T3S genes and/or overexpression of *hrcN* cannot compensate for the loss of HrcQ. This is in contrast to the finding that the phenotype of flagellar C ring mutants can be partially complemented by overexpression of the ATPase-encoding gene *fliI* or by upregulation of the regulatory genes *flhDC* [40], [41]. The C ring of flagellar T3S systems is therefore probably not essential for secretion, which is different from the crucial role of HrcQ from *X. campestris* pv. *vesicatoria*.

Loss of pathogenicity of the *hrcQ* deletion mutant was specifically caused by the absence of HrcQ because the mutant phenotype was partially complemented by ectopic expression of *hrcQ in trans*. Overexpression of *hrcQ* under control of the *lac* promoter did not complement the *hrcQ* mutant phenotype and significantly reduced T3S and pathogenicity of *hrcQ* wild-type strains. Surprisingly, *lac* promoter-driven expression of *hrcQ* also affected bacterial growth *in vitro*, suggesting that elevated HrcQ levels are detrimental for bacterial fitness (Fig. 1). The latter observation was unexpected because the *in vitro* growth of *X. campestris* pv. *vesicatoria* is assumed to be independent of the T3S system. The negative effect of *hrcQ* overexpression on bacterial growth was less pronounced in complex medium, suggesting that it can be partially compensated by favourable environmental conditions. To circumvent the dominant-negative effects caused by increased HrcQ amounts, we inserted *hrcQ* under control of the native promoter into the *hpaFG* region next to the *hrp* gene cluster of *X. campestris* pv. *vesicatoria hrcQ* deletion mutants. Chromosomally encoded HrcQ-c-Myc fully complemented the phenotype of *hrcQ* deletion mutants with respect to plant reactions, which confirms the above hypothesis that increased copy numbers of *hrcQ* are not favourable for pathogenicity (Fig. 3).

Given the homology of HrcQ with predicted C ring components from animal pathogenic bacteria, we assumed that HrcQ is a cytoplasmic component of the T3S system. In line with this hypothesis, fractionation studies revealed that HrcQ is predominantly present in cytoplasmic fractions and specifically associates with the bacterial membranes under T3S-permissive conditions (Fig. 4). This is reminiscent of the membrane association of the HrcQ homologs HrcQ_A_ and HrcQ_B_ from *Pseudomonas syringae* that are translated as two separate proteins [56]. Notably, we have previously shown that also the cytoplasmic ATPase HrcN and its predicted regulator HrcL from *X. campestris* pv. *vesicatoria* specifically associate with the bacterial membranes under T3S-activating conditions [20]. Our *in vitro* interaction studies showed that both HrcN and HrcL interact with HrcQ (Fig. 5), suggesting that all three proteins are present in a complex which associates with the membrane-spanning components of the T3S system under secretion-permissive conditions. This is in agreement with previously reported interactions between predicted C ring components and the ATPase complex of T3S systems from animal pathogenic bacteria [32], [57]-[60]. Our interaction studies also revealed a self-interaction of HrcQ, suggesting that HrcQ can form homo-oligomers as is expected for a predicted C ring component. Complex formation was also described for HrcQ_A_ and HrcQ_B_ from *P. syringae* and the two translation products of *yscQ* from *Yersinia* [56], [61].

In addition to HrcN and HrcL, HrcQ interacts with the IM proteins HrcU and HrcV that are essential components of the membrane-spanning export apparatus (Fig. 5) [14]. HrcU contains four transmembrane helices and a cytoplasmic domain (HrcU_C_) that is proteolytically cleaved and interacts with the T3S4 protein HpaC [26], [45], [62]. HrcU_C_ is presumably involved in the T3S substrate specificity switch and in substrate binding because it interacts with the early T3S substrate HrpB2 [21], [26]. As HrcU also interacts with HrcQ (Fig. 5) and the ATPase HrcN (via the cytoplasmic domain) as well as with the predicted regulator of the ATPase, HrcL (via the transmembrane region) [20], it is presumably not only required for the control of substrate specificity but additionally contributes to the docking of the predicted HrcQ-HrcL-HrcN complex to the secretion apparatus at the IM.

In contrast to HrcU, the IM protein HrcV has not yet been intensively characterized in *X. campestris* pv. *vesicatoria*. While previous studies revealed an interaction of HrcV with the general T3S chaperone HpaB and the T3S4 protein HpaC, it is yet unknown whether HrcV can directly bind to secreted proteins as was shown for HrcV homologs from flagellar T3S systems (FlhA protein family) [18], [63]-[66]. The new finding of an interaction between the cytoplasmic domain of HrcV and HrcQ suggests that HrcV is involved in the recruitment of cytoplasmic components of the T3S system as is postulated for HrcU. An interaction between HrcV and the ATPase complex still needs to be investigated.

Notably, HrcQ also interacts with the T3S4 protein HpaC, which might be involved in the targeting of T3S substrates to the secretion system (Fig 6). As HpaC additionally binds to the ATPase HrcN as well as to the IM proteins HrcV and HrcU [18], [20], it is possible that the T3S apparatus contains multiple binding sites for HpaC. In agreement with this hypothesis, T3S substrates or chaperone-substrate complexes from animal pathogenic bacteria interact with the predicted C ring as well as with the ATPase and/or the cytoplasmic domains of members of the YscV family of IM proteins [27], [32], [33], [46], [63], [65], [67]-[70]. In future studies, it has to be investigated whether the interaction of HpaC with HrcQ is essential for the T3S substrate specificity switch. Notably, additional interaction studies showed that HrcQ also interacts with different T3S substrates including the early substrate HrpB2 and effector proteins (Fig. 6). This was not caused by unspecific binding because HrcQ did not significantly interact with GST, GST-HpaB and GST-XopA. Given the interaction of HrcQ with secretion substrates, HrcQ is (besides HrcU_C_) the second known potential substrate binding site of the T3S system from *X. campestris* pv. *vesicatoria*. As the early T3S substrate HrpB2 interacts with both HrcQ and HrcU_C_, it is possible that the T3S apparatus provides several binding sites for a given secretion substrate. In future studies we will investigate whether the binding of HrpB2 and other T3S substrates to HrcQ is required for their efficient secretion.

The recruitment and/or secretion of T3S substrates by the T3S system is assumed to depend on an N-terminal secretion signal that is not conserved on the amino acid level [71], [72]. T3S signals have been localized in several T3S substrates from plant and animal pathogenic bacteria and presumably are required for the targeting and docking of these protein to the secretion apparatus. Notably, however, in most cases the contribution of the T3S signal to substrate docking has not yet been analyzed. We have previously shown that the T3S signal of the TAL effector AvrBs3 is located within the N-terminal 50 amino acids that also provide a binding site for HpaB [17]. Surprisingly, however, pull-down assays with HrcQ and AvrBs3 derivatives revealed that the N-terminal region of AvrBs3 is dispensable for the efficient interaction with HrcQ (Fig. 6), suggesting that the T3S signal from AvrBs3 is not required for the binding to HrcQ. This indicates that the interaction of AvrBs3 with HrcQ occurs after the T3S signal-mediated targeting of AvrBs3 to the T3S system. It remains to be investigated how the T3S signal is recognized and whether it is required for substrate binding.

In summary, we conclude from our findings that HrcQ is likely an important substrate docking site of the T3S system but is presumably not the primary binding site for secreted proteins.

## Supporting Information

Figure S1 Complementation studies with strain 85*Δ*hrcQ. X. campestris* pv. *vesicatoria* strains 85* (wt) and 85*Δ*hrcQ* (Δ*hrcQ*) carrying plasmid pBRM (-) or derivatives thereof expressing *hrcQ* or *hrcQ-c-myc* under control of the native (*pnat*) or the *lac* (*plac*) promoter as indicated were inoculated at a density of 4×10^7^ CFU ml^-1^ into leaves of susceptible ECW and resistant ECW-10R pepper plants. Disease symptoms were photographed 9 dpi. For the better visualization of the HR, leaves were bleached in ethanol 2 dpi. Dashed lines mark the infiltrated areas.
(TIF)

Figure S2 Ectopic expression of *hrcN-c-myc* does not complement the *hrcQ* mutant phenotype. (A) Infection studies with derivatives of strain 85*Δ*hrcQ.* Strains 85-10 (wt), 85* (wt), 85-10Δ*hrcQ* (Δ*hrcQ*) and 85*Δ*hrcQ* (Δ*hrcQ*) carrying plasmid pBRM (-) or encoding HrcN-c-Myc as indicated were inoculated at a density of 4×10^8^ CFU ml^-1^ into leaves of susceptible ECW and resistant ECW-10R pepper plants. Disease symptoms were photographed 9 dpi. For the better visualization of the HR, leaves were bleached in ethanol 2 dpi. Dashed lines mark the infiltrated areas. Expression of *hrcN-c-myc* under control of the *lac* promoter was previously shown to complement the *hrcN* mutant phenotype [20]. (B) HrcN-c-Myc is stably synthesized in strain 85*Δ*hrcQ. X. campestris* pv. *vesicatoria* strains 85* (wt) and 85*Δ*hrcQ* (Δ*hrcQ*) carrying plasmid pBRM (-) or encoding HrcN-c-Myc as indicated were grown in NYG medium and total cell extracts were analyzed by immunoblotting, using a c-Myc epitope-specific antibody.
(TIF)

Figure S3 Generation of the suicide vector pLAND-P. DNA fragments of the *hpaFG* region flanking the *lacZ* gene, the *lac* promoter (*lacP*) and the 3× c-Myc epitope-encoding sequence were cloned into the suicide vector pOK1 (see Materials and Methods). *Bsa*I sites upstream of *lacP* and downstream of *lacZ* allow the directional cloning of genes of interest in frame with the 3× c-Myc epitope-encoding sequence. Genes are represented by arrows. The DNA sequences given in the boxes refer to the overhangs that are generated after restriction of the DNA with *Bsa*I.
(TIF)
