# Supplementary figures and images for: HrcQ Provides a Docking Site for Early and Late Type III Secretion Substrates from Xanthomonas
Source: PLoS One. 2012 Nov 30;7(11):e51063. doi: 10.1371/journal.pone.0051063 (PMC3511370; doi:10.1371/journal.pone.0051063)

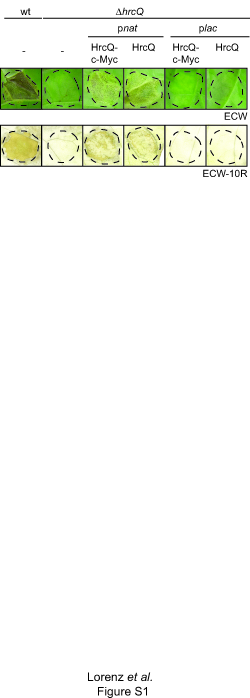

Supplement: Figure S1 — Complementation studies with strain 85*ΔhrcQ. X. campestris pv. vesicatoria strains 85* (wt) and 85*ΔhrcQ (ΔhrcQ) carrying plasmid pBRM (-) or derivatives thereof expressing hrcQ or hrcQ-c-myc under control of the native (pnat) or the lac (plac) promoter as indicated were inoculated at a density of 4×10^7^ CFU ml-1 into leaves of susceptible ECW and resistant ECW-10R pepper plants. Disease symptoms were photographed 9 dpi. For the better visualization of the HR, leaves were bleached in ethanol 2 dpi. Dashed lines mark the infiltrated areas. (TIF) [file pone.0051063.s001.tif]

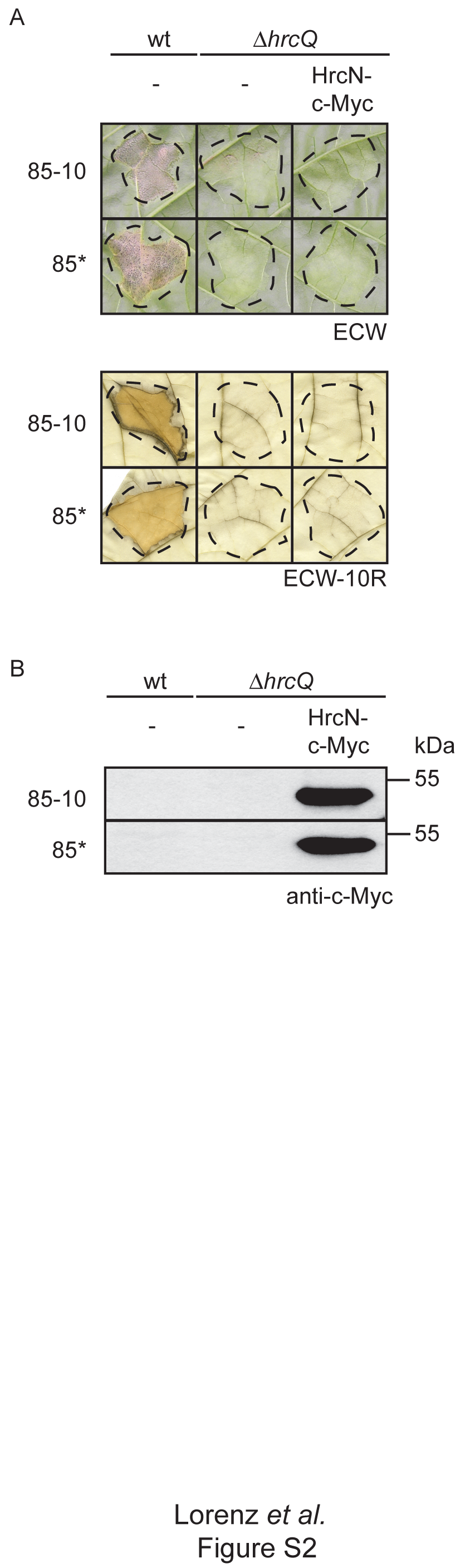

Supplement: Figure S2 — Ectopic expression of hrcN-c-myc does not complement the hrcQ mutant phenotype. (A) Infection studies with derivatives of strain 85*ΔhrcQ. Strains 85-10 (wt), 85* (wt), 85-10ΔhrcQ (ΔhrcQ) and 85*ΔhrcQ (ΔhrcQ) carrying plasmid pBRM (-) or encoding HrcN-c-Myc as indicated were inoculated at a density of 4×10^8^ CFU ml-1 into leaves of susceptible ECW and resistant ECW-10R pepper plants. Disease symptoms were photographed 9 dpi. For the better visualization of the HR, leaves were bleached in ethanol 2 dpi. Dashed lines mark the infiltrated areas. Expression of hrcN-c-myc under control of the lac promoter was previously shown to complement the hrcN mutant phenotype [20]. (B) HrcN-c-Myc is stably synthesized in strain 85*ΔhrcQ. X. campestris pv. vesicatoria strains 85* (wt) and 85*ΔhrcQ (ΔhrcQ) carrying plasmid pBRM (-) or encoding HrcN-c-Myc as indicated were grown in NYG medium and total cell extracts were analyzed by immunoblotting, using a c-Myc epitope-specific antibody. (TIF) [file pone.0051063.s002.tif]

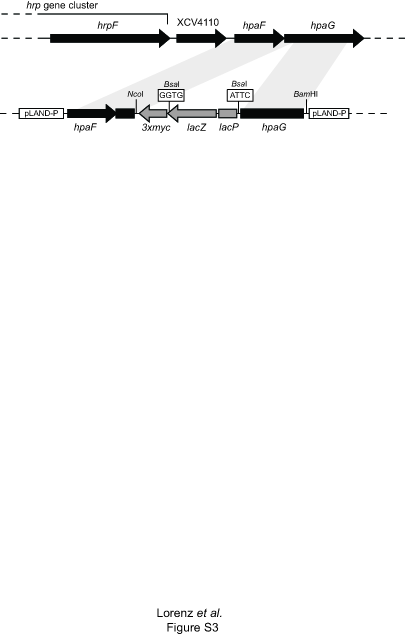

Supplement: Figure S3 — Generation of the suicide vector pLAND-P. DNA fragments of the hpaFG region flanking the lacZ gene, the lac promoter (lacP) and the 3× c-Myc epitope-encoding sequence were cloned into the suicide vector pOK1 (see Materials and Methods). BsaI sites upstream of lacP and downstream of lacZ allow the directional cloning of genes of interest in frame with the 3× c-Myc epitope-encoding sequence. Genes are represented by arrows. The DNA sequences given in the boxes refer to the overhangs that are generated after restriction of the DNA with BsaI. (TIF) [file pone.0051063.s003.tif]
